# Draft Genome Sequence of *Nafulsella turpanensis* ZLM-10^T^, a Novel Member of the Family *Flammeovirgaceae*

**DOI:** 10.1128/genomeA.00263-14

**Published:** 2014-04-03

**Authors:** Lei Zhang, Meiru Si, Lingfang Zhu, Changfu Li, Yahong Wei, Xihui Shen

**Affiliations:** State Key Laboratory of Crop Stress Biology for Arid Areas and College of Life Sciences, Northwest A&F University, Yangling, Shaanxi, People’s Republic of China

## Abstract

*Nafulsella turpanensis* ZLM-10^T^ is a slightly halophilic, Gram-negative, rod-shaped, gliding, pale-pink-pigmented bacterium in the family *Flammeovirgaceae*, and it shows resistance to gentamicin, kanamycin, neomycin, and streptomycin. Here, we report the genome sequence of *N. turpanensis* strain ZLM-10^T^, which has a 4.8-Mb genome and a G+C content of 45.67%.

## GENOME ANNOUNCEMENT

*Nafulsella turpanensis* strain ZLM-10^T^ was originally isolated from the upper soil layer of an arid area in Xinjiang Province, China, and subjected to a polyphasic taxonomic study (1). It is a novel member of the family *Flammeovirgaceae*, exhibiting low levels of 16S rRNA gene sequence similarity (<90.3%) to the type strains of recognized bacterial species (1). The phylogenetically closest relatives of strain ZLM-10^T^ were species of the genera *Cesiribacter* and *Marivirga*, with sequence similarities of 89.1 to 90.2% (1). Strain ZLM-10^T^ grows optimally in the presence of 2% sea salts and shows resistance to gentamicin (10 µg), kanamycin (30 µg), neomycin (30 µg), and streptomycin (10 µg) (1). In this work, *N. turpanensis* strain ZLM-10^T^ was selected for sequencing on the basis of its phylogenetic position and phenotypic features.

The genome of *N. turpanensis* strain ZLM-10^T^ was sequenced on the Illumina HiSeq2000 platform using a 100-bp paired-end library with an insert size of 500 bp. A total of 499.99 Mb of filtered paired-end reads was obtained, representing about 100.14-fold coverage of the genome. All reads were assembled into 135 contigs (109 contigs with >199 bases) and 98 scaffolds by using SOAPdenovo 1.05 (2). Protein-coding sequences were predicted and analyzed by using Glimmer 3.0 (3), and functional annotation of genes was performed by BLASTP searching of the KEGG (4), clusters of orthologous groups (COG) (5), Swiss-Prot (6), TrEMBL (7), and NR (8) databases. rRNA and tRNA genes were detected by using RNAmmer (9) and tRNAscan-SE (10), respectively. Transposons were predicted by RepeatMasker and RepeatProteinMasker (11), and tandem repeat sequences were analyzed by Tandem Repeats Finder (12). The draft genome, excluding the gaps, has a total of 4,809,301 bases with an average G+C content of 45.67%. A total of 4,698 predicted coding sequences (CDSs), 3 rRNA genes, and 43 tRNA genes were identified; 21,219-bp transposable elements and 21,730-bp tandem repeats were predicted, accounting for 0.44% and 0.45% of the genome, respectively. Among the 4,698 genes identified, only 407 were classified into 32 certain functional KEGG sets.

Among all the members of the family *Flammeovirgaceae*, only *Marivirga tractuosa* H-43^T^ was sequenced, and the complete genome sequence has been published (13). *Marivirga tractuosa* H-43^T^ has a smaller genome (4.5 Mb), a significantly lower G+C content (35.5%), and fewer predicted CDSs (3,808) than *Nafulsella turpanensis* ZLM-10^T^. These results indicate the large genomic differences between *Nafulsella* and *Marivirga*. Therefore, the genome sequence of *Nafulsella turpanensis* ZLM-10^T^ will be helpful for understanding a new branch of the family *Flammeovirgaceae* in the phylum *Bacteroidetes*.

### Nucleotide sequence accession numbers.

This whole-genome shotgun project has been deposited at DDBJ/EMBL/GenBank under the accession number ANNU00000000. The version described in this paper is the first version, ANNU01000000.
